# Supplementary material for: The bacterial toxin ExoU requires a host trafficking chaperone for transportation and to induce necrosis
Source: Nat Commun. 2021 Jun 29;12:4024. doi: 10.1038/s41467-021-24337-9 (PMC8241856; doi:10.1038/s41467-021-24337-9)
Supplement: Supplementary file 1 — Supplementary information [file 41467_2021_24337_MOESM1_ESM.pdf]

Supplementary Information

The bacterial toxin ExoU requires a host trafficking chaperone for transportation and to induce necrosis

Vincent Deruelle[1], Stéphanie Bouillot[1], Viviana Job[1], Emmanuel Taillebourg[2], Marie-Odile Fauvarque[2], Ina Attrée[1] and Philippe Huber[1]*

*Corresponding author: phuber@cea.fr

Supplementary Table 1: Strains and plasmids

Names	Relevant characteristics	Reference/Source
***E. coli* strains**		
One Shot™ TOP10 Chemically Competent *E. coli*	F- *mcrA* Δ(*mrr-hsd*RMS-*mcr*BC) Φ80*lac*ZΔM15 Δ *lac*X74 *rec*A1 *ara*D139 Δ(*araleu*)7697 *gal*U *gal*K *rps*L (StrR) *end*A1 *nup*G	Invitrogen, C404003
NEB® 5-alpha Competent *E. coli* (High Efficiency)	*fhuA2 Δ(argF-lacZ)U169 phoA glnV44 Φ80 Δ(lacZ)M15 gyrA96 recA1 relA1 endA1 thi-1 hsdR17*	NEB, C2987H
Lucigen Endura electrocompetent cells	*recA13 supE44 ara-14 galK2 lacY1 proA2 rpsL20(StrR) xyl-5 λ– leu mtl-1 F– mcrB mrr hsdS20(rB–, mB–)*	Biosearch Technologies, 60242
Quick Change XL1-Blue Supercompetent cells	*recA1 endA1 gyrA96 thi-1 hsdR17 supE44 relA1 lac [F´ proAB lacIqZΔM15 Tn10]*	Agilent, 200519-4
Stbl3™ Chemically Competent *E. coli*	F-*mcr*B *mrrhsd*S20(rB-, mB-) *rec*A13 *sup*E44 *ara*-14 *gal*K2 *lac*Y1 *pro*A2 *rps*L20(StrR) *xyl*-5 λ-*leumtl*-1	Invitrogen, C737303
***P. aeruginosa* strains**		
PA14	ExoU+	[1]
PP34	ExoU+	[2]
IHMA879472	$T3SS^-$ and encodes ExlB-ExlA two-partner system	[3]
PA14Δ*exoU*	$ExoU^-$	[4]
PP34Δ*exoU*	$ExoU^-$	This study
PP34Δ*exoU::exoU*^{S142A}-*bla*	PP34Δ*exoU* strain expressing ExoU^{S142A}-Bla fusion	This study
CHAΔ*exoSexoT*	$ExoS^-$ and $ExoT^-$	[5]
CHAΔ*exoSexoT* ::*exoU*^{S142A}(CHA-*exoU*^{S142A})	$ExoS^-$ and $ExoT^-$ and $ExoU^+$ catalytically inactivated; Cb^R	[6]
CHAΔ*exoSexoT* ::*exoU*$^{S142A-K178R}$(CHA-*exoU*$^{S142A-K178R}$)	CHAΔ*exoSexoT* expressing ExoU$^{S142A-K178R}$	[6]
CHAΔ*exoT*	$ExoT^-$ and $ExoS^+$	[5]
**Bacterial vectors**		
pIA*exoS-bla*	Contains *exoS* gene downstream of the β-lactamase gene ; Cb^R	[7]
pIA*exoU*^{S142A}-*bla*	pIA*exoS-bla* plasmid where *exoS* gene is excised and replaced by *exoU*^{S142A}; Cb^R	This study

pUCP*ExoU*^{S142A}*spcU*	Contains *exoU* gene which is catalytically inactivated and *spcU* gene ; Cb^{R}	[6]
pUC57-*DNAJC5-FLAG*	Contains *DNAJC5* gene with N-terminal FLAG-tag; Amp^{R}	This study
pUC57-*DNAJC5*$^{S10A\text{-}S34A}$-*FLAG*	Contains FLAG-tagged *DNAJC5* gene mutated on phosphorylation sites ; Amp^{R}	This study
pUC57-*DNAJC5*^{H43Q}-*FLAG*	Contains FLAG-tagged *DNAJC5* gene mutated on the Hsc70/Hsp70 binding site; Amp^{R}	This study
pUC57-*DNAJC5*^{L115R}-*FLAG*	Contains FLAG-tagged *DNAJC5* gene with L115R mutation in the cysteine-string domain ; Amp^{R}	This study
pUC57-*DNAJC5*$^{\Delta L116}$-*FLAG*	Contains FLAG-tagged *DNAJC5* gene with the deletion of L116 in the cysteine-string domain ; Amp^{R}	This study
pUC57-*DNAJC5*$^{\Delta Jdomain}$-*FLAG*	Contains FLAG-tagged *DNAJC5* gene with the deletion of the J-domain ; Amp^{R}	This study

Mammalian vectors

psPAX2	Lentiviral packaging plasmid	Addgene, 12260
pMD2.G	VSV-G envelope expressing plasmid	Addgene, 12259
pLentiCRISPRv2	Lentiviral vector containing *Cas9* nuclease gene, Puro^{R}	
pLVX-*IRES-neo*	Lentiviral vector for bicistronic expression of a gene together with a neomycin-resistance marker	Clonetech, 632181
pLentiCRISPRv2-gRNA-*DNAJC5*	pLentiCRISPRv2 with the gRNA targeting *DNAJC5* gene; Puro^{r}	This study
pLVX-*DNAJC5-FLAG*	pLVX-*IRES-neo* vector which contains FLAG-tagged *DNAJC5* gene; Neo^{R}	This study
pLVX-*DNAJC5*$^{S10A\text{-}S34A}$-*FLAG*	pLVX-*IRES-neo* vector which contains FLAG-tagged *DNAJC5* gene mutated on phosphorylation sites; Neo^{R}	This study
pLVX-*DNAJC5*^{H43Q}-*FLAG*	pLVX-*IRES-neo* vector which contains FLAG-tagged *DNAJC5* gene mutated on Hsc70/Hsp70 binding site; Neo^{R}	This study
pLVX-*DNAJC5*^{L115R}-*FLAG*	pLVX-*IRES-neo* vector which contains FLAG-tagged *DNAJC5* gene with L115R mutation in the cysteine-string domain; Neo^{R}	This study
pLVX-*DNAJC5*$^{\Delta L116}$-*FLAG*	pLVX-*IRES-neo* vector which contains FLAG-tagged *DNAJC5* gene with the deletion of L116 in the cysteine-string domain; Neo^{R}	This study

pLVX-*DNAJC5*$^{\Delta Jdomain}$-*FLAG*	pLVX-*IRES-neo* vector which contains FLAG-tagged *DNAJC5* gene with the deletion of the J-domain; Neo^R	This study
DNAJC5-GFP	Mammalian expression plasmid for DNAJC5 with N-terminal GFP tag	[8]

Supplementary Table 2: Oligonucleotides for PCR amplification

Names	Primers (5' → 3’)
ExoU-Gm_Fw	GTCCGGCTCCGGAGTCAC
ExoU-Gm_Rev	GCTGCAGCATTTCGCGCG
ExoU-BamHI	CCGGATCCCAAGGCGCTTGATCAGTGG
ExoU-XbaI	GGTCTAGATGTGAACTCCTTATTCCGCCAAG
DNAJC5-Fw	GGAGTGCTGGGATGACAGG
DNAJC5-Rev	CAGTCCCTGGGATCTACGG

Supplementary Table 3: List of primers used to amplify and sequence the gRNA-containing cassettes of each sample after the ExoU screen

Names	Primers (5' → 3’)*
Uninfected_Fw	AATGATACGGCGACCACCGAGATCTACACATAGAGGCACACTCTTTCCCTACACGACGCTCTTCCGATCTTTGTGGAAAGGACGAAACACCG
Uninfected_Rev	CAAGCAGAAGACGGCATACGAGATTCTCCGGAGTGACTGGAGTTCAGACGTGTGCTCTTCCGATCTACTTGCTATTTCTAGCTCTAAAAC
ExoU-infected-1_Fw	AATGATACGGCGACCACCGAGATCTACACTATAGCCTACACTCTTTCCCTACACGACGCTCTTCCGATCTTTGTGGAAAGGACGAAACACCG
ExoU-infected-1_Rev	CAAGCAGAAGACGGCATACGAGATTTCTGAATGTGACTGGAGTTCAGACGTGTGCTCTTCCGATCTACTTGCTATTTCTAGCTCTAAAAC
ExoU-infected-2_Fw	AATGATACGGCGACCACCGAGATCTACACTATAGCCTACACTCTTTCCCTACACGACGCTCTTCCGATCTTTGTGGAAAGGACGAAACACCG
ExoU-infected-2_Rev	CAAGCAGAAGACGGCATACGAGATACGAATTCGTGACTGGAGTTCAGACGTGTGCTCTTCCGATCTACTTGCTATTTCTAGCTCTAAAAC
ExoU-infected-3_Fw	AATGATACGGCGACCACCGAGATCTACACTATAGCCTACACTCTTTCCCTACACGACGCTCTTCCGATCTTTGTGGAAAGGACGAAACACCG
ExoU-infected-3_Rev	CAAGCAGAAGACGGCATACGAGATAGCTTCAGGTGACTGGAGTTCAGACGTGTGCTCTTCCGATCTACTTGCTATTTCTAGCTCTAAAAC

*Red sequences denote i5 or i7 index. Blue sequences denote annealing sequence on TKOv3 vector

Supplementary Table 4: Oligonucleotides to knock-out the *DNAJC5* gene

Names	Primers (5' → 3’)*
gRNA-DNAJC5_Fw	**CACCG**GAGGCCGCAGAAGACAAACA
gRNA-DNAJC5_Rev	**AAAC**TGTTTGTCTTCTGCGGCCTC**C**

* Bold letters represent overhangs for cloning in *Bsm*BI-digested plentiCRISPRv2

Supplementary Table 5: Synthetic *DNAJC5-FLAG* gene sequence and its derivatives

Names	Sequence (5' → 3')*
DNAJC5-FLAG	GAATTCATGGACTACAAAGACCATGACGGCGATTATAAAGATCATGACA<br>TCGATTACAAGGATGACGATGACAAGGCAGACCAGAGACAGCGCTCAC<br>TGTCTACCTCTGGGGAGTCATTGTACCACGTCCTTGGGTTGGACAAGAA<br>CGCAACCTCAGATGACATTAAAAAGTCCTATCGGAAGCTTGCCTTGAAAT<br>ATCACCCCGACAAGAACCCCGACAACCCGGAGGCCGCGGACAAGTTTAA<br>GGAGATCAACAACGCGCACGCCATCCTCACGGACGCCACAAAAAGGAA<br>CATCTACGACAAGTACGGCTCGCTGGGTCTCTACGTGGCCGAGCAGTTT<br>GGGGAAGAGAACGTGAACACCTACTTCGTGCTGTCCAGCTGGTGGGCC<br>AAGGC**T**CTGTT**C**GT**G**TT**T**TGCGGCCTCCTCACGTGCTGCTACTGCTGCTG<br>CTGTCTGTGCTGCTGCTTCAACTGCTGCTGCGGGAAGTGTAAGCCCAAG<br>GCGCCTGAAGGCGAGGAGACGGAGTTCTACGTGTCCCCCGAGGATCTG<br>GAGGCACAGCTGCAGTCTGACGAGAGGGAGGCCACAGACACGCCGATC<br>GTCATACAGCCGGCATCCGCCACCGAGACCACCCAGCTCACAGCCGACT<br>CCCACCCCAGCTACCACACTGACGGGTTCAACTAAGGATCC
DNAJC5-FLAG$^{S10A-S34A}$	GAATTCATGGACTACAAAGACCATGACGGCGATTATAAAGATCATGACA<br>TCGATTACAAGGATGACGATGACAAGGCAGACCAGAGACAGCGCTCAC<br>TGGCTACCTCTGGGGAGTCATTGTACCACGTCCTTGGGTTGGACAAGAA<br>CGCAACCTCAGATGACATTAAAAAGGCCTATCGGAAGCTTGCCTTGAAA<br>TATCACCCCGACAAGAACCCCGACAACCCGGAGGCCGCGGACAAGTTTA<br>AGGAGATCAACAACGCGCACGCCATCCTCACGGACGCCACAAAAAGGA<br>ACATCTACGACAAGTACGGCTCGCTGGGTCTCTACGTGGCCGAGCAGTT<br>TGGGGAAGAGAACGTGAACACCTACTTCGTGCTGTCCAGCTGGTGGGC<br>CAAGGC**T**CTGTT**C**GT**G**TT**T**TGCGGCCTCCTCACGTGCTGCTACTGCTGCT<br>GCTGTCTGTGCTGCTGCTTCAACTGCTGCTGCGGGAAGTGTAAGCCCAA<br>GGCGCCTGAAGGCGAGGAGACGGAGTTCTACGTGTCCCCCGAGGATCT<br>GGAGGCACAGCTGCAGTCTGACGAGAGGGAGGCCACAGACACGCCGAT<br>CGTCATACAGCCGGCATCCGCCACCGAGACCACCCAGCTCACAGCCGAC<br>TCCCACCCCAGCTACCACACTGACGGGTTCAACTAAGGATCC
DNAJC5-FLAG^{H43Q}	GAATTCATGGACTACAAAGACCATGACGGCGATTATAAAGATCATGACA<br>TCGATTACAAGGATGACGATGACAAGGCAGACCAGAGACAGCGCTCAC<br>TGTCTACCTCTGGGGAGTCATTGTACCACGTCCTTGGGTTGGACAAGAA<br>CGCAACCTCAGATGACATTAAAAAGTCCTATCGGAAGCTTGCCTTGAAAT<br>ATCAGCCCGACAAGAACCCCGACAACCCGGAGGCCGCGGACAAGTTTA<br>AGGAGATCAACAACGCGCACGCCATCCTCACGGACGCCACAAAAAGGA<br>ACATCTACGACAAGTACGGCTCGCTGGGTCTCTACGTGGCCGAGCAGTT<br>TGGGGAAGAGAACGTGAACACCTACTTCGTGCTGTCCAGCTGGTGGGC<br>CAAGGC**T**CTGTT**C**GT**G**TT**T**TGCGGCCTCCTCACGTGCTGCTACTGCTGCT<br>GCTGTCTGTGCTGCTGCTTCAACTGCTGCTGCGGGAAGTGTAAGCCCAA<br>GGCGCCTGAAGGCGAGGAGACGGAGTTCTACGTGTCCCCCGAGGATCT<br>GGAGGCACAGCTGCAGTCTGACGAGAGGGAGGCCACAGACACGCCGAT<br>CGTCATACAGCCGGCATCCGCCACCGAGACCACCCAGCTCACAGCCGAC<br>TCCCACCCCAGCTACCACACTGACGGGTTCAACTAAGGATCC

* Bold letters represent the mutations to prevent the Cas9 endonuclease from cleaving the gene. Red letters represent the S10A, S34A and H43Q mutations. Green letters are *Eco*RI and *Bam*HI restriction sites. Yellow letters denote the 3X-*FLAG* sequence.

Supplementary Table 6: Oligonucleotides for site-directed mutagenesis

Names	Primers (5' → 3')*
DNAJC5_L115R_F	CTGTTCGTGTTTTGCGGCCGCCTCACGTGCTGCTACTGC
DNAJC5_L115R_R	GCAGTAGCAGCACGTGAGGCGGCCGCAAAACACGAACAG
DNAJC5_ΔL116_F	CTGTTCGTGTTTTGCGGCCTCACGTGCTGCTACTGCTGCTGC
DNAJC5_ΔL116_R	GCAGCAGCAGTAGCAGCACGTGAGGCCGCAAAACACGAACAG
DNAJC5_ΔJdomain_F	CCAGAGACAGCGCTCACTGTCTACCGGTCTCTACGTGGCCGAGCAGTTTGG
DNAJC5_ΔJdomain_R	CCAAACTGCTCGGCCACGTAGAGACCGGTAGACAGTGAGCGCTGTCTCTGG

* Red letters represent the mutated or inserted nucleotides

Supplementary Table 7: Number of independent repetitions for each experiment

Figures	Number of independent experiments	Supplementary figures	Number of independent experiments
1c	3	1b	1
1d	2	1c	4
1e	2	1d	1
1f	3	1e	4
1g	3	2a	1
1h	2	2b	2
2b	4	2c	1
2c	4	3b	2
3a	2	3c	3
3b	2	4b	1
3c	4	4c	2
3d	2	4d	1
4b	4	5	2
4c	2	6a	1
4d	1	6b	1
4e	3	7	2
5a	2		
5b	2		
5c	6		
5d	2		
5e	4		
6	3		

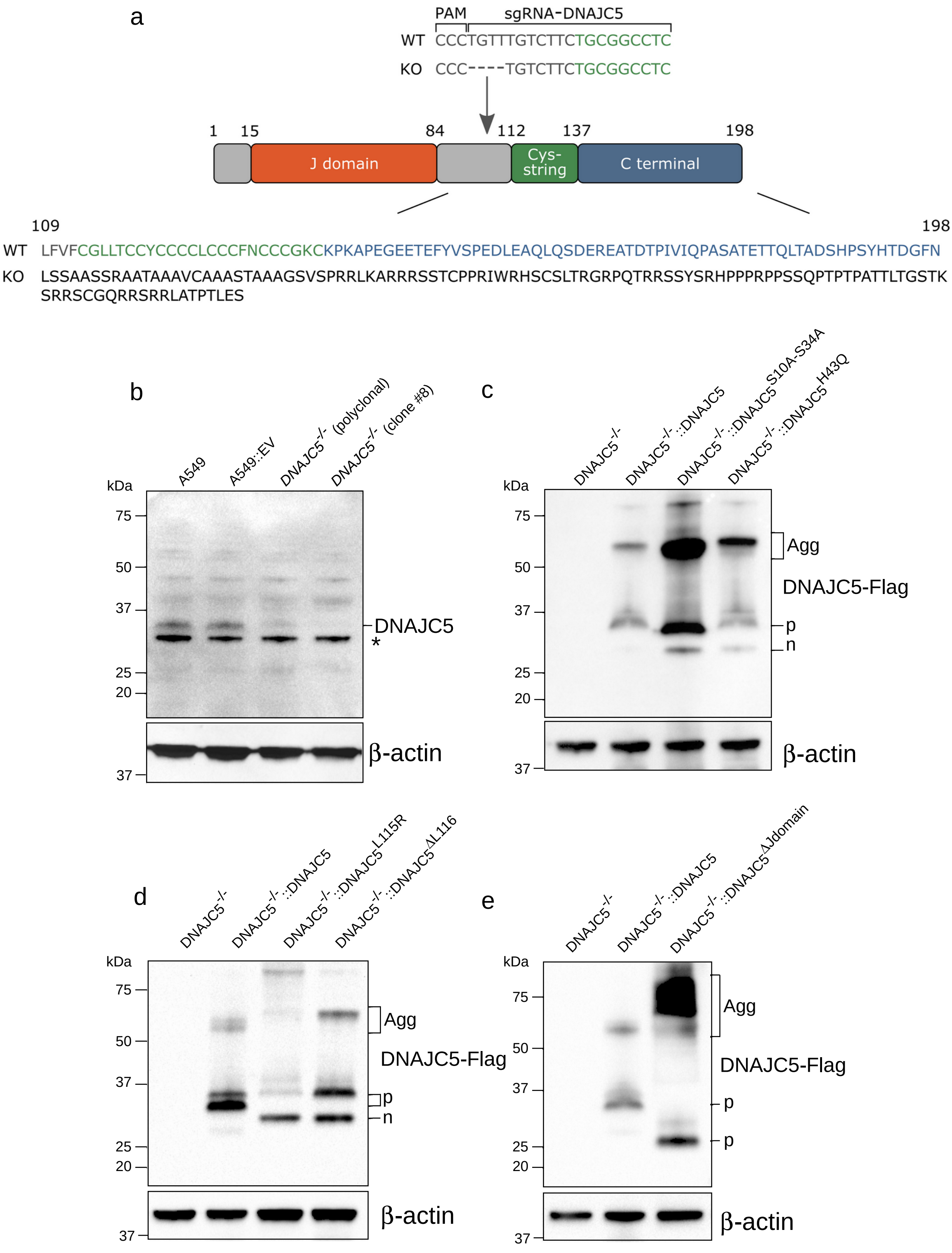
a
PAM
sgRNA-DNAJC5
WT CCCTGTTTGTCTTCTGCGGCCTC
KO CCC----TGTCTTCTGCGGCCTC
1 15 84 112 137 198
J domain
Cys-string
C terminal
109
198
WT LFVFCGLLTCCYCCCCLCCCFNCCCGKCKPKAPEGEETEFYVSPEDLEAQLQSDEREATDTPIVIQPASATETTQLTADSHPSYHTDGFN
KO LSSAASSRAATAAAVCAAASTAAAGSVSPRRLKARRRSSTCPPRIWRHSCSLTRGRPQTRRSSYSRHPPPRPPSSQPTPTPATTLTGSTK SRRSCGQRRSRRLATPTLES
b
A549
A549::EV
DNAJC5-/- (polyclonal)
DNAJC5-/- (clone #8)
kDa
75
50
37
25
20
37
DNAJC5
*
β-actin
c
DNAJC5-/-
DNAJC5-/- ::DNAJC5
DNAJC5-/- ::DNAJC5S10A-S34A
DNAJC5-/- ::DNAJC5H43Q
kDa
75
50
37
25
20
37
Agg
DNAJC5-Flag
p
n
β-actin
d
DNAJC5-/-
DNAJC5-/- ::DNAJC5
DNAJC5-/- ::DNAJC5L115R
DNAJC5-/- ::DNAJC5ΔL116
kDa
75
50
37
25
20
37
Agg
DNAJC5-Flag
p
n
β-actin
e
DNAJC5-/-
DNAJC5-/- ::DNAJC5
DNAJC5-/- ::DNAJC5ΔJdomain
kDa
75
50
37
25
20
37
Agg
DNAJC5-Flag
p
p
β-actin

Supplementary Fig. 1. Sequence of DNAJC5- gene clone #8 and expression of wild-type and mutant DNAJC5.

a. Part of the native DNAJC5 sequence (WT), aligned with the mutated sequence present in the selected DNAJC5$^{-/-}$ cells clone #8. The four missing nucleotides are indicated. The same deletion was present on both alleles. The PAM sequence and the sequence of the gRNA used for inactivation are shown. The protein sequence of mutated DNAJC5 from amino-acid 109 is indicated below. **b.** Western blot with cellular extracts from A549, A549::EV and DNAJC5$^{-/-}$ (polyclonal population and clone#8) cells, revealed with DNAJC5 antibodies. β-actin was used as loading control. **c.** Western blot showing DNAJC5-FLAG expression in DNAJC5$^{-/-}$::DNAJC5, DNAJC5$^{-/-}$::DNAJC5^{H43Q} and DNAJC5$^{-/-}$::DNAJC5$^{S10A-S34A}$ cells, revealed with the FLAG antibodies. **d.** Western blot showing DNAJC5-FLAG expression in DNAJC5$^{-/-}$::DNAJC5, DNAJC5$^{/-}$::DNAJC5^{L115R} and DNAJC5$^{-/-}$::DNAJC5$^{\Delta L116}$ cells, revealed with the FLAG antibodies. **e.** Western blot showing DNAJC5-FLAG expression in DNAJC5$^{-/-}$::DNAJC5, DNAJC5$^{-/}$::DNAJC5$^{\Delta Jdomain}$ cells, revealed with FLAG antibodies. Abbreviations: (n) native and (p) palmitoylated forms of DNAJC5; Agg: aggregates. (*) represents a non-specific band only visible in highly exposed images. Source data are provided as a Source Data file.

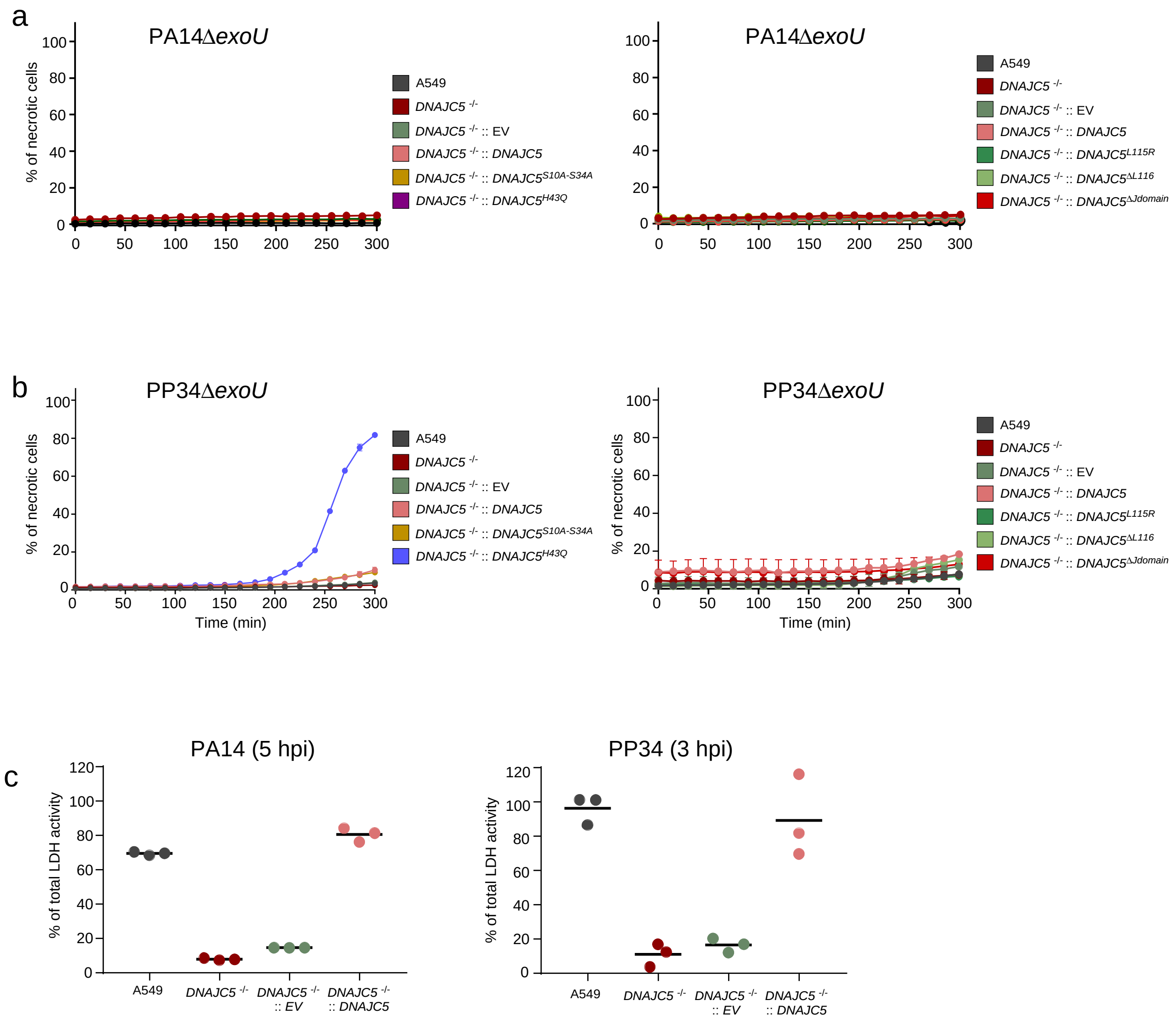

Supplementary Fig. 2. Controls of toxicity experiments using ExoU isogenic mutants.

a, b. Necrotizing activity of PA14Δ*exoU* (**a**) and PP34Δ*exoU* (**b**) was assayed on A549, DNAJC5$^{-/-}$ and complemented strains by PI incorporation kinetics, as in Fig. 1 (n = 3 for all). **c.** Necrotizing activity of PA14 and PP34 on A549, DNAJC5$^{-/-}$, DNAJC5$^{-/-}$::EV and DNAJC5$^{-/-}$::DNAJC5 cells was also assayed by measuring LDH release at indicated times (n = 3). Source data are provided as a Source Data file.

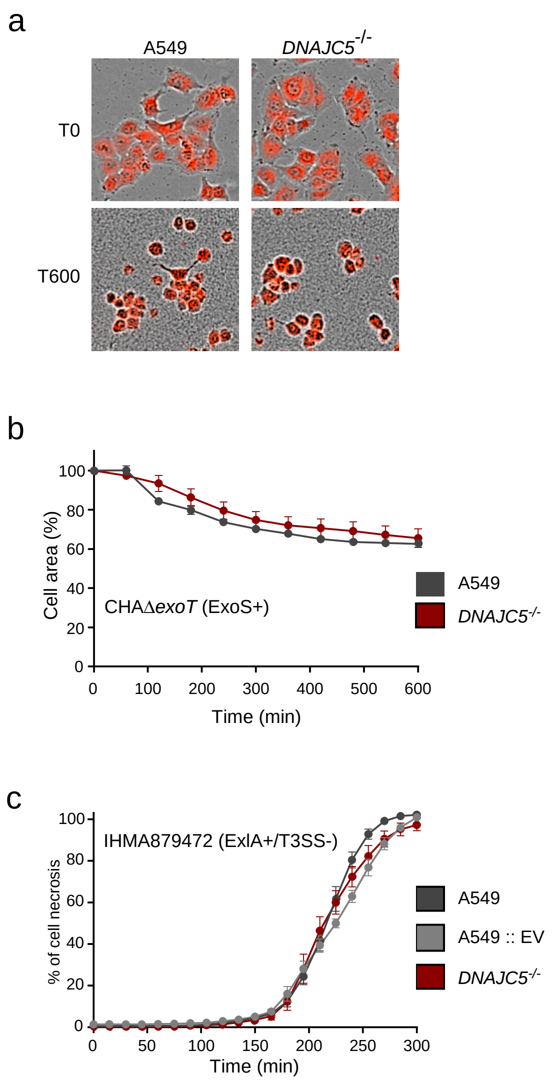

Supplementary Fig. 3. DNAJC5 is not required for ExoS or ExlA toxicity.

a. ExoS-dependent cytotoxicity. A549 cells or DNAJC5$^{-/-}$ cells loaded with CellTracker Red CMTPX were infected with the CHA*ΔexoT* strain expressing ExoS. Intoxication was followed by time-lapse microscopy (n = 6). Images show merged acquisitions of phase contrast and CellTracker fluorescence at the beginning (T0) and at 10 hpi (T600). Both cell types displayed ExoS-dependent cell rounding. **b.** Quantification of ExoS-induced cell rounding using ImageJ software. **c.** A549, A549 ::EV and DNAJC5$^{-/-}$ cells were infected with the ExlA+ IHMA879472 strain, and cell necrosis was monitored based on PI incorporation (n = 4). EV, empty vector. Source data are provided as a Source Data file.

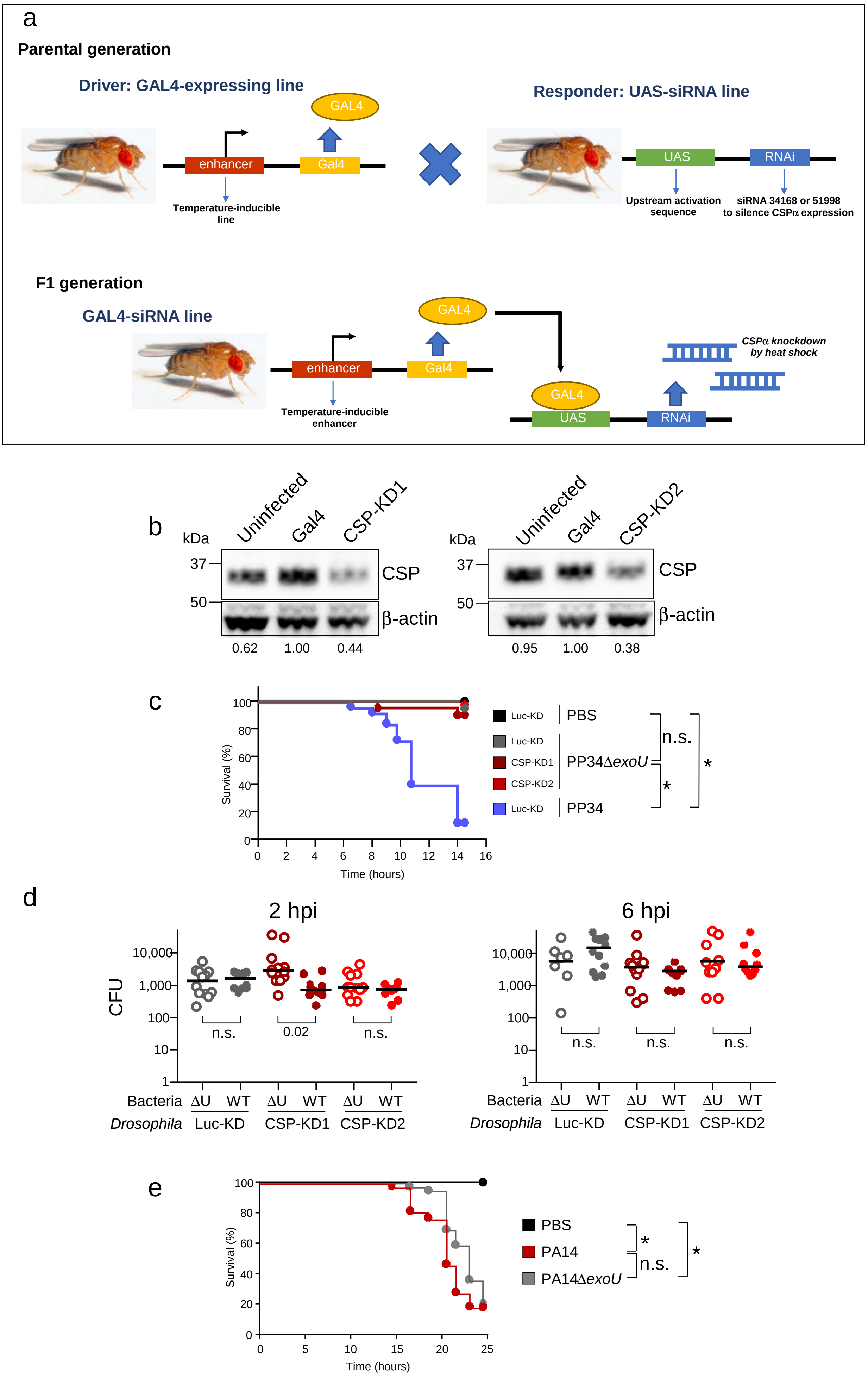
a
Parental generation
Driver: GAL4-expressing line
enhancer
Gal4
GAL4
Temperature-inducible line
Responder: UAS-siRNA line
UAS
RNAi
Upstream activation sequence
siRNA 34168 or 51998 to silence CSPα expression
F1 generation
GAL4-siRNA line
enhancer
Gal4
GAL4
Temperature-inducible enhancer
GAL4
UAS
RNAi
CSPα knockdown by heat shock
b
kDa
Uninfected
Gal4
CSP-KD1
37
50
CSP
β-actin
0.62
1.00
0.44
kDa
Uninfected
Gal4
CSP-KD2
37
50
CSP
β-actin
0.95
1.00
0.38
c
Survival (%)
Time (hours)
Luc-KD PBS
Luc-KD
CSP-KD1 PP34ΔexoU
CSP-KD2
Luc-KD PP34
n.s.
*
*
d
2 hpi
6 hpi
CFU
10,000
1,000
100
10
1
n.s.
0.02
n.s.
n.s.
n.s.
n.s.
Bacteria ΔU WT ΔU WT ΔU WT
Drosophila Luc-KD CSP-KD1 CSP-KD2
Bacteria ΔU WT ΔU WT ΔU WT
Drosophila Luc-KD CSP-KD1 CSP-KD2
e
Survival (%)
Time (hours)
PBS
PA14
PA14ΔexoU
*
n.s.
*

Supplementary Fig. 4. Production of *Csp* knockdown in *Drosophila* and survival assay.
a. Mating scheme to generate CSP-KD flies. **b.** Western blots showing CSP levels in flies using two different RNAi transgenes (CSP-KD1 and CSP-KD2). β-actin was used as loading control. CSP/β-actin signal ratios are indicated below each lane. **c**. Survival of flies (Luc-KD, CSP-KD and CSP-KD2) infected as in Fig. 2 with PP34Δ*exoU* strain was recorded and data are represented as Kaplan-Meyer curves, (n = 19; 20 and 37 , respectively). As controls, Luc-KD flies were infected with PP34 (n = 26) or were mock-infected with PBS (n = 15). Statistical differences were established using the Log-Rank test. Differences were not significant (n.s.) between curves representing PP34Δ*exoU* infections and PBS; Differences were significant (*, $p < 0.001$) between PP34Δ*exoU* infection curves and PP34 infection curve, as well as between PBS and PP34. **d**. The colony-forming units (CFU) of bacteria present in infected flies were assayed at 2 and 6 h.p.i.. Results are shown for individual flies (n = 9-12, as shown) together with the median (bar). Statistical differences between flies infected with PP34Δ*exoU* and PP34 were calculated with a two-sided Mann-Whitney's test and are shown in the figure. n.s., not significant. **e**. Survival curves of Gal4 *Drosophila* infected with PA14 (n = 43), PA14 Δ*exoU* (n = 39) or mock-infected with PBS (n = 27). Statistical differences were established using the Log-Rank test. Differences were not significant between PA14 and PA14Δ*exoU*. Significant differences (*,$p < 0.001$) were calculated between PA14 or PA14Δ*exoU* and PBS curves. Source data are provided as a Source Data file.

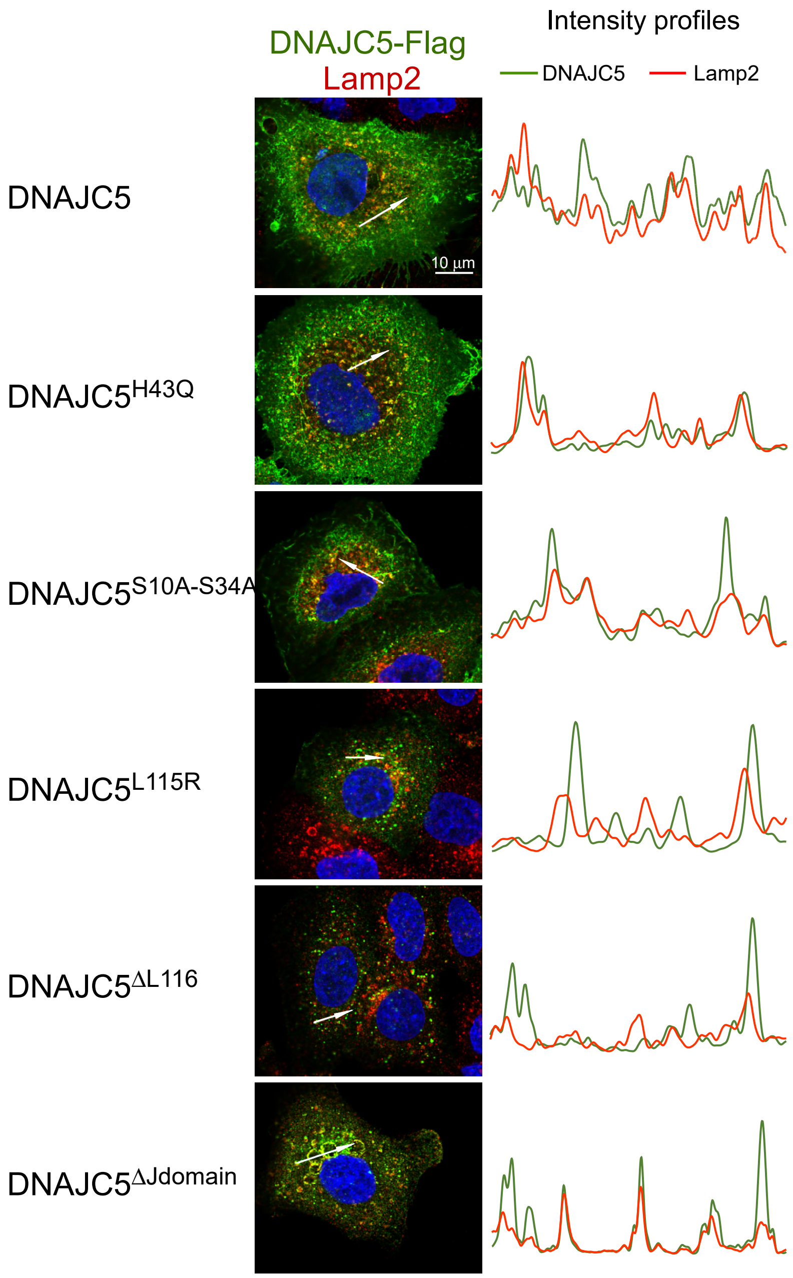

Supplementary Fig. 5. Partial colocalization of DNAJC5 (wild type or mutants) with Lamp2.

Immunostaining to reveal colocalization of DNAJC5 (green) and Lamp2 (red) in DNAJC5$^{-/-}$ cells complemented with DNAJC5-FLAG or a mutated form of DNAJC5, as indicated. Arrows indicate where the intensity profiles for both labels were measured. Intensity profiles are shown on the right.

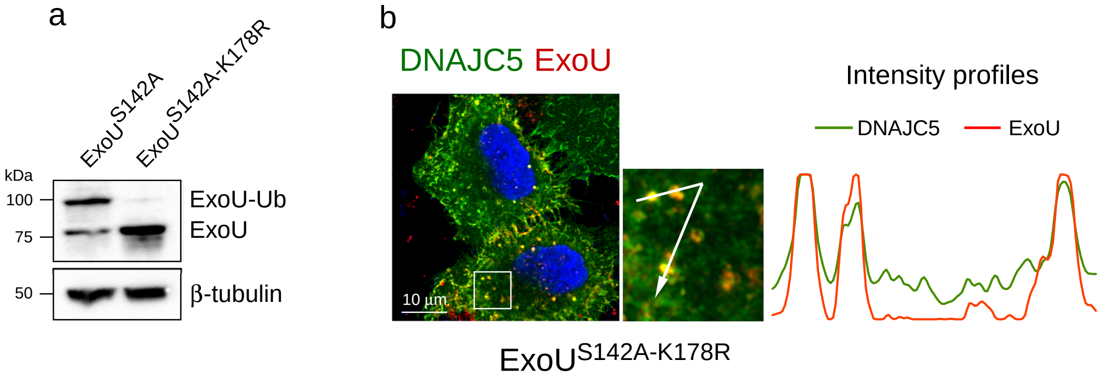

Supplementary Fig. 6. ExoU ubiquitylation is not required for ExoU binding to DNAJC5+ vesicles.

a. Western blot showing the absence of ubiquitylation of $ExoU^{K178R}$ in A549 cells infected with CHA-$exoU^{S142A\text{-}K178R}$. Source data are provided as a Source Data file. **b.** Immunostaining revealing colocalization of DNAJC5-FLAG (green) and ExoU (red) in DNAJC5::DNAJC5 cells infected with CHA-$exoU^{S142A\text{-}K178R}$. The arrow indicates where the intensity profiles for both labels were measured. Intensity profiles are shown on the right.

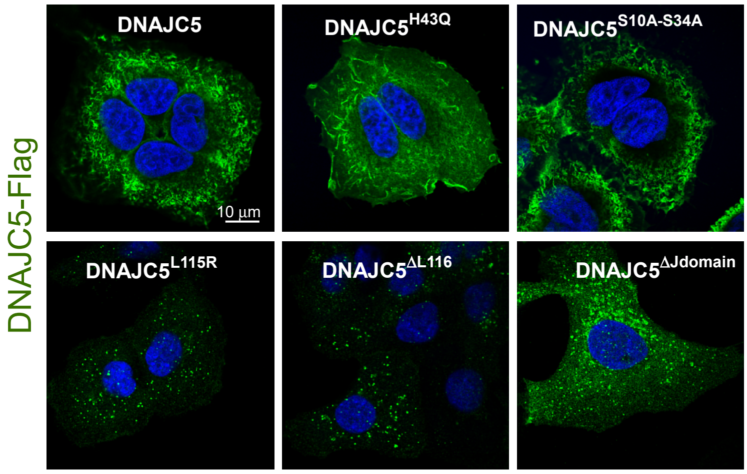

Supplementary Fig. 7. Expression and localization of DNAJC5 mutants.

DNAJC5 and derivatives, fused to a FLAG tag, were expressed in DNAJC5$^{-/-}$ cells. Cells were fixed and labelled with an anti-FLAG antibody. One z-section image is shown for each cell line.

References

1 Rahme, L. G. *et al.* Use of model plant hosts to identify Pseudomonas aeruginosa virulence factors. *Proc Natl Acad Sci U S A* **94**, 13245-13250, doi:10.1073/pnas.94.24.13245 (1997).

2 Berthelot, P. *et al.* Genotypic and phenotypic analysis of type III secretion system in a cohort of Pseudomonas aeruginosa bacteremia isolates: evidence for a possible association between O serotypes and exo genes. *The Journal of infectious diseases* **188**, 512-518, doi:10.1086/377000 (2003).

3 Kos, V. N. *et al.* The resistome of Pseudomonas aeruginosa in relationship to phenotypic susceptibility. *Antimicrob Agents Chemother* **59**, 427-436, doi:10.1128/AAC.03954-14 (2015).

4 Liberati, N. T. *et al.* An ordered, nonredundant library of Pseudomonas aeruginosa strain PA14 transposon insertion mutants. *Proc Natl Acad Sci U S A* **103**, 2833-2838, doi:10.1073/pnas.0511100103 (2006).

5 Quenee, L., Lamotte, D. & Polack, B. Combined sacB-based negative selection and cre-lox antibiotic marker recycling for efficient gene deletion in pseudomonas aeruginosa. *BioTechniques* **38**, 63-67, doi:10.2144/05381ST01 (2005).

6 Gendrin, C. *et al.* Structural basis of cytotoxicity mediated by the type III secretion toxin ExoU from Pseudomonas aeruginosa. *PLoS pathogens* **8**, e1002637, doi:10.1371/journal.ppat.1002637 (2012).

7 Verove, J. *et al.* Injection of Pseudomonas aeruginosa Exo toxins into host cells can be modulated by host factors at the level of translocon assembly and/or activity. *PLoS One* **7**, e30488, doi:10.1371/journal.pone.0030488 (2012).

8 Greaves, J. *et al.* Palmitoylation-induced aggregation of cysteine-string protein mutants that cause neuronal ceroid lipofuscinosis. *J Biol Chem* **287**, 37330-37339, doi:10.1074/jbc.M112.389098 (2012).
