# Supplementary material for: The bacterial toxin ExoU requires a host trafficking chaperone for transportation and to induce necrosis
Source: Nat Commun. 2021 Jun 29;12:4024. doi: 10.1038/s41467-021-24337-9 (PMC8241856; doi:10.1038/s41467-021-24337-9)
Supplement: Supplementary file 3 — Reporting Summary [file 41467_2021_24337_MOESM3_ESM.pdf]

nature research

Corresponding author(s): Philippe HUBER

Last updated by author(s): Jun 9, 2021

Reporting Summary

Nature Research wishes to improve the reproducibility of the work that we publish. This form provides structure for consistency and transparency in reporting. For further information on Nature Research policies, see our Editorial Policies and the Editorial Policy Checklist.

Statistics

For all statistical analyses, confirm that the following items are present in the figure legend, table legend, main text, or Methods section.

n/a	Confirmed	
☐	☒	The exact sample size (*n*) for each experimental group/condition, given as a discrete number and unit of measurement
☐	☒	A statement on whether measurements were taken from distinct samples or whether the same sample was measured repeatedly
☐	☒	The statistical test(s) used AND whether they are one- or two-sided<br>*Only common tests should be described solely by name; describe more complex techniques in the Methods section.*
☒	☐	A description of all covariates tested
☐	☒	A description of any assumptions or corrections, such as tests of normality and adjustment for multiple comparisons
☐	☒	A full description of the statistical parameters including central tendency (e.g. means) or other basic estimates (e.g. regression coefficient) AND variation (e.g. standard deviation) or associated estimates of uncertainty (e.g. confidence intervals)
☐	☒	For null hypothesis testing, the test statistic (e.g. *F*, *t*, *r*) with confidence intervals, effect sizes, degrees of freedom and *P* value noted<br>*Give P values as exact values whenever suitable.*
☒	☐	For Bayesian analysis, information on the choice of priors and Markov chain Monte Carlo settings
☒	☐	For hierarchical and complex designs, identification of the appropriate level for tests and full reporting of outcomes
☒	☐	Estimates of effect sizes (e.g. Cohen's *d*, Pearson's *r*), indicating how they were calculated

Our web collection on statistics for biologists contains articles on many of the points above.

Software and code

Policy information about availability of computer code

Data collection	CRISPR-AnalyzeR 1.50, a web-based analysis platform for pooled CRISPR Screens
Data analysis	Model-based Analysis of Genome-wide CRISPR-Cas9 Knockout (MAGeCK) to identify important genes from CRISPR-Cas9 screens and GraphPad 7.04 for all other statistical analyses. ImageJ 1.49o and Icy 2.1.4.0 for image analysis.

For manuscripts utilizing custom algorithms or software that are central to the research but not yet described in published literature, software must be made available to editors and reviewers. We strongly encourage code deposition in a community repository (e.g. GitHub). See the Nature Research guidelines for submitting code & software for further information.

Data

Policy information about availability of data

All manuscripts must include a data availability statement. This statement should provide the following information, where applicable:

- Accession codes, unique identifiers, or web links for publicly available datasets
- A list of figures that have associated raw data
- A description of any restrictions on data availability

The sequencing data generated in this study have been deposited in the NCBI Gene Expression Omnibus (GEO) and are accessible through GEO accession number GSE154751 https://www.ncbi.nlm.nih.gov/geo/query/acc.cgi?acc=GSE154751.

Field-specific reporting

Please select the one below that is the best fit for your research. If you are not sure, read the appropriate sections before making your selection.

- [x] Life sciences
- [ ] Behavioural & social sciences
- [ ] Ecological, evolutionary & environmental sciences

For a reference copy of the document with all sections, see nature.com/documents/nr-reporting-summary-flat.pdf

Life sciences study design

All studies must disclose on these points even when the disclosure is negative.

Sample size	For genomic screen, we used the sample size of a previously reported study: Blondel et al., 2016, Cell Host & Microbe 20, 226–237, as well as indications from Moffat's lab, as indicated in Methods. Briefly, the screen was performed with three biological replicates. Averaged reads per gRNA was 34 in the uninfected condition, yielding sufficient representativness for statistical analysis. For cytotoxicity studies or cell retraction assay, analyses of 1 field in at least 3 replicates were sufficient to yield high statistical significance. For Bla activity assay, 3 replicates for each condition provides a 80% power using a two-sided alpha of 0.05 to detect a difference of 15% with SD 0.1 using a t-test, between conditions using ExoU-bla. For fly infection, 5 groups of 10 flies per condition is a standard size for survival studies, as previously established (Fauvarque et al. Microb Pathog 2002, 32:287). For PLA2 activity test, 3 replicates for each condition provides a 90% power using a two-sided alpha of 0.05 to detect a difference of 10% with SD 80 using a one-way ANOVA. For CFU comparison, 12 replicates for each condition provides a 80% power using a alpha of 0.05 to detect a difference of 30% with SD 2,000 using a two-sided t-test (based on Fauvarque et al. Microb Pathog 2002, 32:287)
Data exclusions	Flies found dead one hour after injection were excluded.
Replication	A list of repetition for each experiment is provided in Supplementary Table 7. All repetitions yielded similar results.
Randomization	Flies were randomly assigned to experimental groups.
Blinding	Lethality of infected flies was evaluated by blinded staff. Blinding was not required for Western blot, cytotoxicity assays, PLA2 dosage and CFU counting because the investigator cannot influence the results. The observation of labelled cells by microscopy and the choice of the images were performed by a group of investigators to minimize subjectivity.

Reporting for specific materials, systems and methods

We require information from authors about some types of materials, experimental systems and methods used in many studies. Here, indicate whether each material, system or method listed is relevant to your study. If you are not sure if a list item applies to your research, read the appropriate section before selecting a response.

Materials & experimental systems

n/a	Involved in the study	
[ ]	[x]	Antibodies
[ ]	[x]	Eukaryotic cell lines
[x]	[ ]	Palaeontology and archaeology
[ ]	[x]	Animals and other organisms
[x]	[ ]	Human research participants
[x]	[ ]	Clinical data
[x]	[ ]	Dual use research of concern

Methods

n/a	Involved in the study	
[x]	[ ]	ChIP-seq
[x]	[ ]	Flow cytometry
[x]	[ ]	MRI-based neuroimaging

Antibodies

Antibodies used	The rabbit polyclonal antibody targeting human CSPα (DNAJC5) was purchased from ThermoFisher (#PA1-776). The mouse polyclonal antibodies against β-actin (#A1978), β-tubulin (#T0198) and FLAG (#F1804) were purchased from Sigma-Aldrich. Specific antisera for ExoU were obtained in rabbits with 50 µg of purified full length recombinant ExoU. The specific antibodies were affinity-purified on His6-ExoU. The mouse monoclonal antibody targeting CSP in Drosophila melanogaster (named DCSP-1) was purchased from DSBH (#ab49). The monoclonal mouse Hsc70 (HSPA8) and Hsp70 (HSPA1A) antibodies were purchased from R&D Systems (#MAB4148 and #MAB1663-SP, respectively). The anti-Lamp2 antibody was from BD Transduction Laboratories (#555803).
Validation	Validation and references for commercial antibodies are provided by the suppliers:<br>Human CSPα, https://www.thermofisher.com/antibody/product/CSP-alpha-Antibody-Polyclonal/PA1-776<br>β-actin, https://www.sigmaaldrich.com/catalog/product/sigma/a1978<br>β-tubulin, https://www.sigmaaldrich.com/catalog/product/sigma/t0198<br>FLAG, https://www.sigmaaldrich.com/catalog/product/sigma/f1804<br>Drosophila CSP, https://dshb.biology.uiowa.edu/DCSP-1-ab49<br>Hsc70, https://www.rndsystems.com/products/human-mouse-rat-hspa8-hsc71-antibody-521503_mab4148

Hsp70, https://www.rndsystems.com/products/human-mouse-rat-hsp70-hspa1a-antibody-242707_mab1663
Lamp-2, https://www.bdbiosciences.com/eu/applications/research/intracellular-flow/intracellular-antibodies-and-isotype-controls/anti-human-antibodies/purified-mouse-anti-human-cd107b-h4b4/p/555803

The ExoU antibody was validated by Western blots using protein extracts from PP34 (ExoU+) and ExoU isogenic mutant strains.

Eukaryotic cell lines

Policy information about cell lines

Cell line source(s)	The human embryonic kidney (HEK) 293T and A549 cells were obtained from ATCC (#CRL-11268 and #CCL-185, respectively). HUVECs were obtained from umbilical cords and used until passage 5.
Authentication	A549 cells were routinely checked for the presence of junctional E-cadherin by immunostaining. HUVECs were checked for the presence of junctional VE-cadherin by immunostaining. HEK cells were not authenticated.
Mycoplasma contamination	A549 and HEK cells were routinely tested by PCR for the absence of mycoplasma and were negative. HUVECs were not tested
Commonly misidentified lines (See ICLAC register)	No commonly misidentified cell lines were used

Animals and other organisms

Policy information about studies involving animals; ARRIVE guidelines recommended for reporting animal research

Laboratory animals	Drosophila melanogaster, CSP-KD1 (Stock #34168 from the Vienna Drosophila Resource Center) and CSP-KD2 (Stock #33645 from the Bloomington Drosophila Stock Center) expressing a shRNA or a long dsRNA targeting Csp, respectively, under the control of GAL4-responsive elements were used. These flies were crossed with transgenic flies expressing the Gal4 gene under the control of a temperature-inducible promoter (hs-Gal4, stock #2077 from the Bloomington Drosophila Stock Center). Flies expressing a siRNA targeting the firefly Luciferase gene (Stock #31603 from the Bloomington Drosophila Stock Center) were also crossed with the heat shock-Gal4 line and used as control. Seven-ten day-old flies were used for the experiment.
Wild animals	This study did not involved wild animals
Field-collected samples	This study did not involve sample collection from the fields
Ethics oversight	No ethical approval is required for Drosophila

Note that full information on the approval of the study protocol must also be provided in the manuscript.
